# Supplementary material for: Patterns of Intron Gain and Loss in Fungi
Source: PLoS Biol. 2004 Nov 30;2(12):e422. doi: 10.1371/journal.pbio.0020422 (PMC532390; doi:10.1371/journal.pbio.0020422)
Supplement: Table S1 — Also available at http://genes.mit.edu/NielsenEtAl/. (4.3 MB ZIP). [file pbio.0020422.st001.zip › NielsenEtAl/html/56.html]

AN7143.1.NCU03009.1.MG10565.1.FG02785.1


```
 CLUSTAL W (1.82) Multiple Sequence Alignments - Introns Inserted


Sequence 1: MG10565.1	444 aa
Sequence 2: FG02785.1	444 aa
Sequence 3: NCU03009.1	445 aa
Sequence 4: AN7143.1	447 aa
Alignment Length: 450 aa
Number Identitical Residues: 256 aa
Alignment Score (without introns) 12342


MG10565.1 	MATATIVKKPLPALTSGWSAEKDFKPLGKLT--KATERSIEPVGPHFLAHARR~SRHKRT
NCU03009.1	--MTTVVPLPLPTLPEGWTAEKDFKPIGKIAQNEATQRTIEPVGPHFLAHARR~ARHKRT
FG02785.1 	MASVATVTLPLPALPSGWAAEKDFKAVGKLS--EATQRSIEPVGPHFLAHARR~ARHKRT
AN7143.1  	MSSAQVVELTLPALPAGWSADKEFKAVGKLS--AATQRNLEPVGPHFLAHARR0KRHHRT
          	 : .  *  .**:*. **:*:*:**.:**::   **:*.:*************  **:**

MG10565.1 	FSEDDRIQAQERAKKVEDEDDGEISEPEDPMMFQRDAKDWK0NQDHYQVLGLSKYRFKAS
NCU03009.1	FSEDDRIQAQERAKNVEEDNESDLSEPEDPMMLSRDAKDWK0QQDHYKVLGLSKYRWRAT
FG02785.1 	FSEDDRIQAQESAKNVEDGDVSDESEPEDPMMLQREAKDWK0TQDHYKILGISKYRWRAT
AN7143.1  	FSEDERIQAQQNVKKTEEEEDDEISEDEDPMMLQRDAKDWK0SQDHYAVLGLSKYRWRAT
          	****:*****: .*:.*: : .: ** *****:.*:*****  **** :**:****::*:

MG10565.1 	EDQIKRAHRKKVLKHHPDKKAAAG-RVDDDNFFKCIQKATEVLLDPVKRRQFDSVDEKAD
NCU03009.1	EEQIKRAHRKKVLKHHPDKKAAAG-RTEDDNFFKCIQKATEVLLDPIKRRQFDSVDEEAD
FG02785.1 	EDQIKKAHRKKVLKHHPDKKAAQG-RVDDDQFFKCIQKATDVLLDPVKRRQFDSVDEEAE
AN7143.1  	PDQIKRAHRKKVLRHHPDKKAALGDRDENDSFFKCIQKATEILSDPVKRRQFDSVDEAAD
          	 :***:*******:******** *.* ::*.*********::* **:********** *:

MG10565.1 	VEPPTKKQLQKGNYYKLWGNVFKAEARFSKTHPVPQFGGEDASKEQVEEFYNFWYNFDSW
NCU03009.1	VEPPTKKQLQKGNFYKLWGNVFKAEGRFSNTQPVPSFGNENSTREEVENFYNFFYNFDSW
FG02785.1 	VEPPTKKQLQKADYYKAWSKVFKSEARFSKTHPVPSFGSADATKEQVEDFYNFWYNFDSW
AN7143.1  	VEPPSKKETQKGNFYKLWRPVFESEARFSKIQPVPQLGDENSTFEEVNEFYNFWYNFDSW
          	****:**: **.::** *  **::*.***: :***.:*. ::: *:*::****:******

MG10565.1 	RSFEYLDEDVPDDNENRDQKRQMERKNANARKKKKVEDNARLRKLLDDASAGDERIKRFR
NCU03009.1	RSFEYLDEDVPDDSESRDQRRHTERKNLNTRKKRKAEDNARLRKLLDDCSAADERIKKFR
FG02785.1 	RTFEYLDEEVPDDNENRDQKRHQERKNTNARKKRKVEDNARLRKLLDDASAGDERIKRFR
AN7143.1  	RTFEYLDEDVPDDNENRDQKRHVEKKNANARRKRKTEDTARLRRLVDDCAALDERIKKFR
          	*:******:****.*.***:*: *:** *:*:*:*.**.****:*:**.:* *****:**

MG10565.1 	QEANAAKNKKKADKEAAEKKAIEDARLKKEAEEKAAKDAEEKAKADREAGKKAKEAAKNA
NCU03009.1	QEANAAKNKKRLEKEAAEKKAAEEAAAKKAAEEAAAKEAEEKAKADREASKKAKEAAKNA
FG02785.1 	QEANASKNKKRLEREAAEKKAKEDAQAAKEAEEKAAKEAEEKAKVDRESSKKAKEAAKNA
AN7143.1  	KAARADKDKKRLEKEAEAKRIAEEKEKARLEEEQRKKEAEEAAKADREKAKKAKEAAKNA
          	: *.* *:**: ::**  *:  *:    :  **   *:*** **.*** .**********

MG10565.1 	VKKNKRVLKGSVKDANYFAS-GDASPAQVDAVLGDVDLVQAKIDADEIAALAGKLNGL-T
NCU03009.1	VKKNKRVLRGSVKDANYFVE-GDASVATIDAVLGDVELIQAKIDPDEIAALAGKLNNL-K
FG02785.1 	VKKNKRVLKGSVKDANYFAA-GEPTPADIDSVLNDVETIQGKIDPDEIAALAGKLNGL-K
AN7143.1  	AKKNKRVLKGSVKDVNYFAESGEPSAAQVDSVLTDVDLINSKIDNEELASLAERLTAAGK
          	.*******:*****.***. :*:.: * :*:** **: ::.*** :*:*:** :*.  ..

MG10565.1 	VADEIKAVWQGEVKRLVDAGKLKDGEAKSFA-
NCU03009.1	VADEIKAVWNEEAARLVGAGKINAGDLKALSA
FG02785.1 	VADEIKAVWTGETKRLVEAGKLKDGDIKVLA-
AN7143.1  	DAAAVKNVYTEEIKRLVAAGKAKEGEVKFFV-
          	 *  :* *:  *  *** *** : *: * :
```
